# Disparate Metabolic Responses in Mice Fed a High-Fat Diet Supplemented with Maize-Derived Non-Digestible Feruloylated Oligo- and Polysaccharides Are Linked to Changes in the Gut Microbiota

**DOI:** 10.1371/journal.pone.0146144

**Published:** 2016-01-05

**Authors:** Junyi Yang, Laure B. Bindels, Rafael R. Segura Munoz, Inés Martínez, Jens Walter, Amanda E. Ramer-Tait, Devin J. Rose

**Affiliations:** 1 Department of Food Science & Technology, University of Nebraska-Lincoln, Lincoln, NE, United States of America; 2 Department of Agricultural, Food, and Nutritional Science, University of Alberta, Edmonton, AB, Canada; 3 Department of Biological Sciences, University of Alberta, Edmonton, AB, Canada; 4 Department of Agronomy & Horticulture, University of Nebraska-Lincoln, Lincoln, NE, United States of America; National Institute of Agronomic Research, FRANCE

## Abstract

Studies have suggested links between colonic fermentation of dietary fibers and improved metabolic health. The objectives of this study were to determine if non-digestible feruloylated oligo- and polysaccharides (FOPS), a maize-derived dietary fiber, could counteract the deleterious effects of high-fat (HF) feeding in mice and explore if metabolic benefits were linked to the gut microbiota. C57BL/6J mice (n = 8/group) were fed a low-fat (LF; 10 kcal% fat), HF (62 kcal% fat), or HF diet supplemented with FOPS (5%, w/w). Pronounced differences in FOPS responsiveness were observed: four mice experienced cecal enlargement and enhanced short chain fatty acid production, indicating increased cecal fermentation (F-FOPS). Only these mice displayed improvements in glucose metabolism compared with HF-fed mice. Blooms in the gut microbial genera *Blautia* and *Akkermansia* were observed in three of the F-FOPS mice; these shifts were associated with reductions in body and adipose tissue weights compared with the HF-fed control mice. No improvements in metabolic markers or weights were detected in the four mice whose gut microbiota did not respond to FOPS. These findings demonstrate that FOPS-induced improvements in weight gain and metabolic health in mice depended on the ability of an individual’s microbiota to ferment FOPS.

## Introduction

Obesity is associated with a cluster of metabolic disorders including insulin resistance, type 2 diabetes, and cardiovascular disease [1]. These diseases diminish the quality of life for a growing proportion of the world’s population, and there is an urgent need to develop strategies that reduce the prevalence of these diseases. Research during the last decade has clearly demonstrated that the microbial community colonizing the gastrointestinal tract (the gut microbiota) contributes to the pathologies associated with obesity [2–6]. The gut microbiota therefore constitutes a promising therapeutic target for dietary strategies to prevent or treat these diseases [7]. Dietary fiber consumption is one strategy by which the gut microbiota composition can be modulated towards putatively beneficial microbial ecologies. In addition, the fermentation of dietary fiber leads to the synthesis of short chain fatty acids (SCFA), which contribute to the exclusion of detrimental bacteria, inhibit the metabolism of harmful substances, strengthen gut barrier function, and exert beneficial effects on the production of gut hormones. By doing so, SCFA may benefit the host by improving glucose homeostasis and blood lipid profiles as well as reducing body weight and colon cancer risk [8]. Together, these combined effects would likely contribute to long-term prevention of the chronic subclinical inflammation that can develop into metabolic syndrome [9–12].

In a previous study, we identified a hydrothermal process to produce non-digestible feruloylated oligo- and polysaccharides (FOPS) from maize [13]. FOPS are composed of hydrolysates of the hemicellulosic component of maize bran, which is principally a complex heteroxylan comprised of a (1, 4)-linked β-D-xylopyranosyl backbone with single or multi-unit branches consisting of α-L-arabinofuranose, β-D-xylopyranose, β-D-glucuronic acid, and a non-carbohydrate antioxidant, ferulic acid [14]. The multi-unit branches in maize heteroxylan are rare among cereal dietary fibers, as are some of the linkages between sugars on these branches [e.g., (1, 2)- and (1, 3)-linkages between β-D-xylopyranose and α-L-arabinofuranose]. Our previous findings with human fecal *in vitro* fermentations demonstrated that this complex structure is more difficult for the microbiota to ferment compared to the hemicellulosic components of other cereals and thus may contribute to sustained SCFA production [13, 15]. Dietary fibers capable of supporting prolonged saccharolytic bacterial fermentation may help in maintaining beneficial SCFA production in the distal colon, which is low in SCFA and particularly prone to disease [16–18]. Our *in vitro* studies have also shown enhanced SCFA production of FOPS from maize bran, especially butyrate, compared with fructans and FOPS produced from wheat bran. Additionally, because of their high ferulic acid content, fermented FOPS samples possessed high levels of antioxidant activity, which may be beneficial for reducing the damaging effects of free radicals, including nitric oxide, released in the colon during inflammatory responses [19, 20]. Because of these promising *in vitro* results, the objective of this present study was to determine the impact of FOPS consumption in counteracting the deleterious effects of high-fat (HF) feeding in mice and explore if metabolic benefits were linked to FOPS modulating the gut microbiota.

## Materials and Methods

### Production and Composition of FOPS

FOPS from maize bran was produced as previously described [13]. In brief, 150 g of finely milled maize bran (Bunge Milling, Crete, NE) was dispersed in 1.35 L of water in a 2 L high-pressure reactor (Model 4848, Parr, Moline, IL). The slurry was heated to 190°C at the rate of 4°C/min under constant stirring (400 rpm) and then cooled to 80°C using an internal serpentine coil with circulating cold water (ca. 15 min). The slurry was centrifuged at 10,000 × g for 10 min and the supernatant retained. Supernatants from a total of ~25 batches were pooled and loaded into a reverse osmosis system (Model R, GEA Filtration, Hudson, WI, USA) equipped with a membrane (molecular weight cut off: 1000; GE 1207106, GEA Process Engineering Inc., Hudson, WI, USA). The FOPS were circulated with ~75 L of distilled water to separate contaminants (permeate) from FOPS (retentate). Following reverse osmosis, FOPS were freeze-dried (Thermal-Vac Technology Inc., Orange, CA, USA). The freeze-dried material was subsequently analyzed for total carbohydrate, total starch, free monosaccharides, free and esterified ferulic acid, furfural, hydroxymethylfurfural (HMF), and protein as described [13]. FOPS were calculated as the sum of all non-starch polymeric sugars and esterified ferulate [13]. The final FOPS preparation contained 59% FOPS, 16% starch, 3.4% other sugars, 2.9% protein, and 8.8% moisture (S1 Table).

### Experimental Diets and Mouse Experiment

The three diets used in the study were prepared by a commercial provider (Research Diets, New Brunswick, NJ USA): low fat [LF; rodent diet with 10 kcal% fat; D12450K], HF (rodent diet with 62% kcal% fat; D12492), and HF supplemented with 5% FOPS (w/w; S2 Table). FOPS were incorporated into the HF diet at the expense of cellulose. Because the FOPS preparation contained small quantities of starch and protein, the amounts of these compounds in the HF diet formulation were reduced to the extent necessary to match the macronutrient and energy content of the HF control diet. The LF diet was included to confirm metabolic aberrations induced by the HF diet.

Twenty-four eight-week old male C57BL/6J mice were purchased from The Jackson Laboratory (Bar Harbor, Maine, USA). Animals were maintained in an environment with a 14 h light/10h dark cycle and controlled temperature and humidity. Mice were randomly assigned to one of the three dietary treatments (n = 8 mice/group), housed as pairs in individually ventilated cages, maintained on autoclaved bedding, and fed autoclaved water. While acclimating to their environment for one week, all mice were fed a regular autoclaved chow diet (Purina Lab Diets, St. Louis, MO) before starting the dietary interventions, which were provided for eight weeks. Diet replacement and recording of both food intake and body weights were performed weekly.

Feces were collected from individual mice three times during the experiment (weeks 0, 1, and 8) and stored at -80°C until further analysis. After eight weeks of experimental diet feeding, mice were euthanized via CO_2_ asphyxiation. Blood was harvested by cardiac puncture and plasma was collected by centrifugation at 13,000 × g for 3 min at 4°C. Adipose tissues, full ceca, and empty cecal tissues were weighed at the time of collection. All biological samples were snap frozen in liquid nitrogen and then stored at -80°C until analysis. The Institutional Animal Care and Use Committee at the University of Nebraska-Lincoln approved all procedures involving animals (Project ID 817: Microbial Perturbation of Gastrointestinal Homeostasis).

### Intraperitoneal Glucose Tolerance Test

An intraperitoneal glucose tolerance test (ipGTT) was performed after seven weeks of dietary intervention. Mice were fasted in clean cages for 6 h prior to the test. Thirty minutes prior to the test, blood was collected from the tip of the tail to measure fasting glucose concentration using a glucose meter (ACCU-CHEK, Aviva Plus system, Indianapolis, IN, USA). An aliquot of blood was also saved for subsequent insulin analysis via ELISA (Mercodia Insulin ELISA Kit, Uppsala Sweden). At time zero, a glucose solution (20 g/100 mL) was injected into the peritoneal cavity (1 g glucose/kg body weight) [21]. Blood glucose was then measured at 0, 15, 30, 60, 90 and 120 min after glucose injection. An index of insulin resistance was calculated using the formula: [fasting glucose (mg/dL) * fasting insulin (μU/mL)]/405 [22].

### Plasma Lipids and Hormones

Plasma amylin, C-peptide, leptin, and resistin were measured by a multiplex immunoassay (Mouse Metabolic Magnetic Bead Panel Kit; Merck Millipore, Billerica, MA, USA) using a MAGPIX instrument (Luminex Corporation, Austin, TX). Plasma triacylglycerol and total cholesterol were determined using an enzymatic reaction and spectrophotometric detection (Infinity TG/Cholesterol kit; Thermo Electron, Waltham, MA, USA).

### Cecal Short and Branch Chain Fatty Acids

SCFA (acetate, propionate, and butyrate) and branched chain fatty acids (BCFA) (*iso*-butyrate and *iso*-valerate) were quantified by gas chromatography in the cecal contents collected at necropsy [23]. Quantification was done by means of internal calibration with 2-ethyl-butyric acid.

### Characterization of the Fecal Microbiota Composition

DNA was extracted from mouse fecal samples after mechanical and enzymatic bacterial cell lysis as previously described [24]. Microbial composition was assessed at weeks 0, 1, and 8 by 16S rRNA gene tag sequencing (MiSeq; Illumina; San Diego, CA, USA) [25] targeting the V5-V6 region with primer pair 784F (5’-RGGATTAGATACCC-3’) and 1064R (5’-CGACRRCCATGCANCACCT-3’). Initial quality filtering and demultiplexing of the resulting reads was performed with Illumina Software. Next, reads were merged with the merge-Illumina-pairs application, which also removed primers and performed further quality check of the sequences [26]. Subsequently, the UPARSE pipeline [27] was used to process the sequences and perform operational taxonomic unit (OTU) clustering, using a 98% similarity cutoff. Sequences were independently subjected to taxonomic classification for phylum to genus characterization of the fecal microbiome using the RDP MultiClassifier 1.1 from the Ribosomal Database Project [28]. Taxonomic bins were computed as proportions based on the total number of sequences in each sample. α-Diversity was calculated using QIIME [29].

### Statistical Analysis

Results are presented as mean ± SEM except where otherwise indicated. The impact of dietary treatments on body mass, metabolic markers, and gut microbiota α-diversity were analyzed using one-factor ANOVA (diet). Body weight and blood glucose during the ipGTT were analyzed using two-factor repeated measures ANOVA (diet, time). Significant differences for ANOVA models were assessed using Bonferroni’s *post hoc* test. Correlations between bacterial groups and host physiological measurements in the HF- and FOPS-fed mice were calculated using Pearson's coefficients. Microbial taxa that were present at <1% abundance in all mice at all time points were excluded from the analyses. P < 0.05 was used to consider a difference as statistically significant. All data were analyzed using SAS software (version 9.4; SAS Institute, Cary, NC, USA) with the exception of PCA, which was performed using XLSTAT software (Statistical Innovations, Belmont, MA USA).

## Results

### Mice Fed a High-Fat Diet Supplemented with FOPS Experienced Inter-Individual Variation in Fermentation

**Fig 1:**
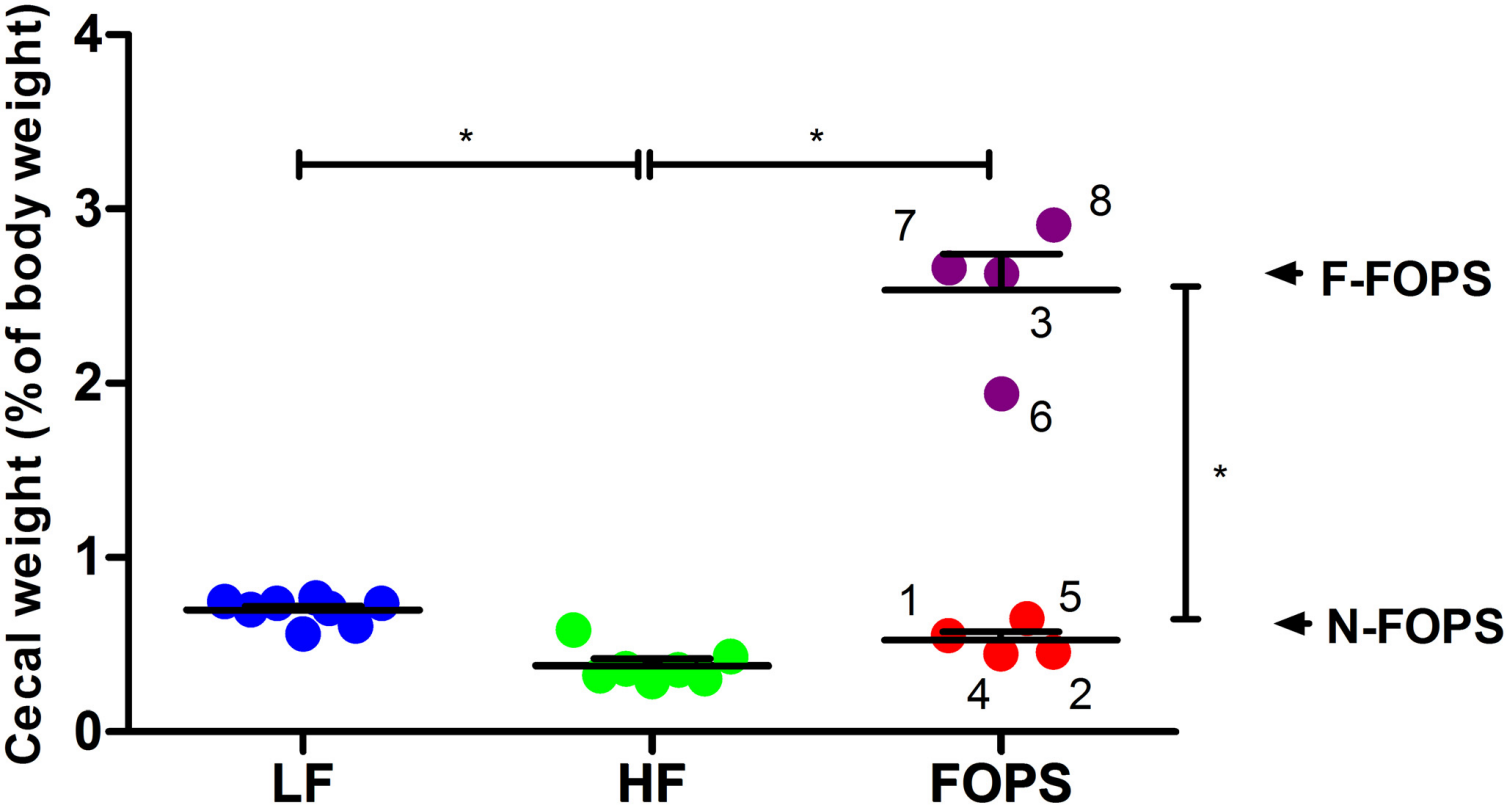
Inter-individual variation in full cecal weights of mice fed a HF diet supplemented with FOPS. Data are presented as mean ± SEM; n = 8 mice/group for LF, HF, and FOPS; n = 4 mice/group for N-FOPS and F-FOPS; mouse numbers are shown for FOPS mice to track individual mice across figures; * indicates a significant difference (p < 0.05) between the indicated treatments using Bonferroni’s multiple comparison test with the following comparisons: LF vs. HF; HF vs. N-FOPS; HF vs. F-FOPS; N-FOPS vs. F-FOPS.

**Fig 2:**
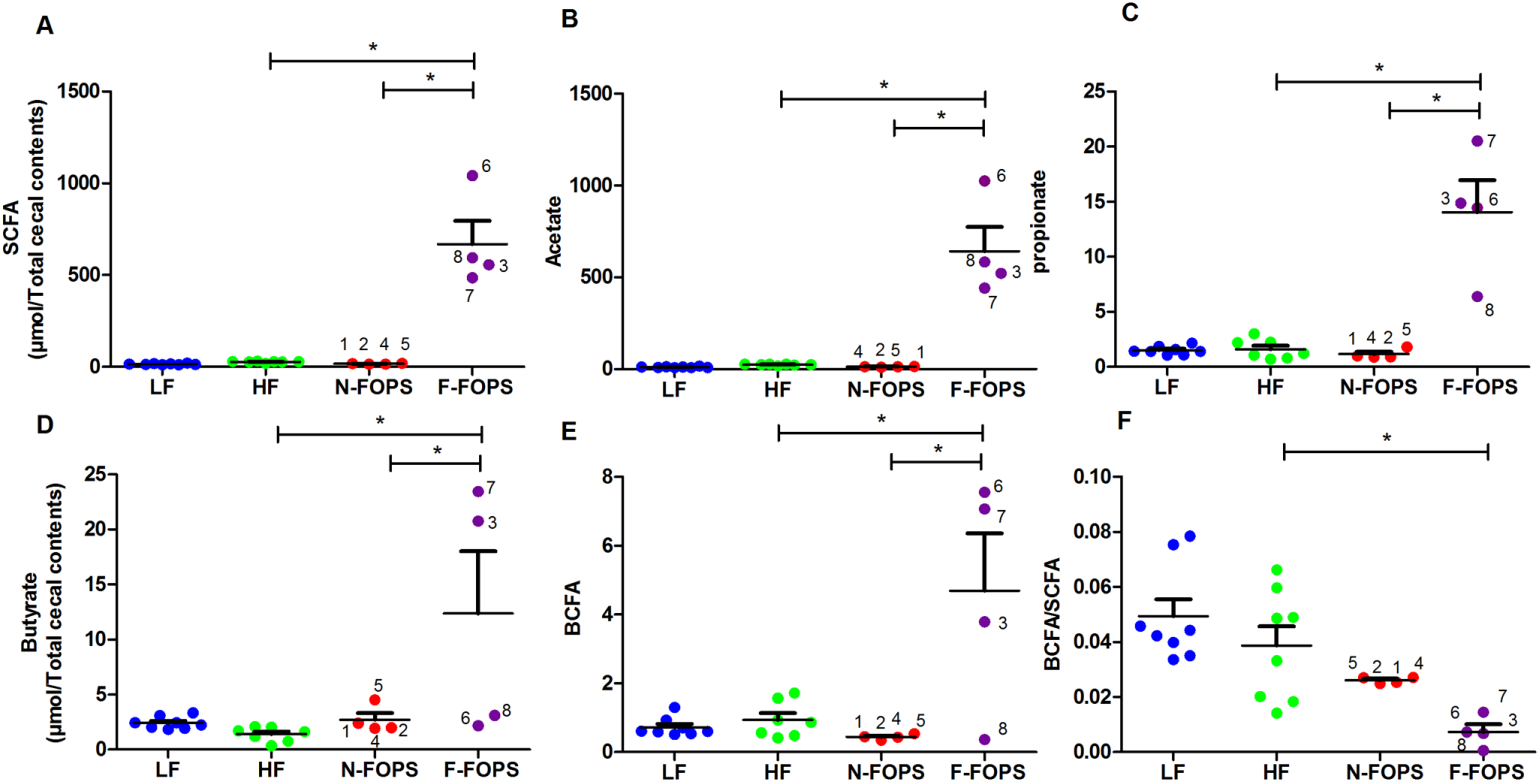
Cecal SCFA in mice after 8 weeks of feeding. SCFA (A), acetate (B), propionate (C), butyrate (D), BCFA (E), SCFA/SCFA ratio (F); data are presented as mean ± SEM; n = 8 mice/group for HF and LF; n = 4 mice/group for N-FOPS and F-FOPS; mouse numbers are shown for FOPS mice to track individual mice across figures; * indicates a significant difference (p < 0.05) between the indicated treatments using Bonferroni’s multiple comparison test with the following comparisons: LF vs. HF; HF vs. N-FOPS; HF vs. F-FOPS; F-FOPS vs. Y-FOPS.

Previous work from our laboratory showed sustained *in vitro* fermentation of non-digestible feruloylated oligo- and polysaccharides (FOPS) from maize by the human fecal microbiota [13]. We observed dramatic cecal enlargement in four mice fed a HF diet supplemented with FOPS, indicative of bacterial fermentation (Fig 1). The other four FOPS fed mice and all of the mice in the other treatment groups contained ceca of normal size. This variation was not due to a cage effect, as two mice with enlarged ceca were from the same cage, while the other two shared cages with mice that had ceca of normal size. The total SCFA pool was significantly elevated in the mice with enlarged ceca compared to all other animals (Fig 2A). These mice also showed marked increases in acetate and propionate pools (Fig 2B and 2C). Butyrate production was elevated in this group as well, but with wide variation among mice (Fig 2D). A significant increase in BCFA production was detected in the mice with enlarged ceca compared with others (Fig 2E); however, the BCFA/SCFA ratio was significantly lower for the mice with enlarged ceca compared to those in other groups (Fig 2F), suggesting that the elevated BCFA production was a result of greater overall fermentative activity and not a tendency toward putrefactive fermentation. No significant differences in cecal SCFA or BCFA pools were evident among LF, HF, and N-FOPS mice (Fig 2). Given that increased cecal weight and elevated SCFA pool were strong indications of increased fermentation and metabolic activity of the gut microbiota [30], we divided the mice on the FOPS diet into those with enlarged ceca that fermented the FOPS (F-FOPS; n = 4) and those with ceca of normal size (N-FOPS; n = 4) for subsequent data analysis and presentation.

### Fermentation of FOPS Was Associated with Limited Weight Gain and White Fat Deposition

**Fig 3:**
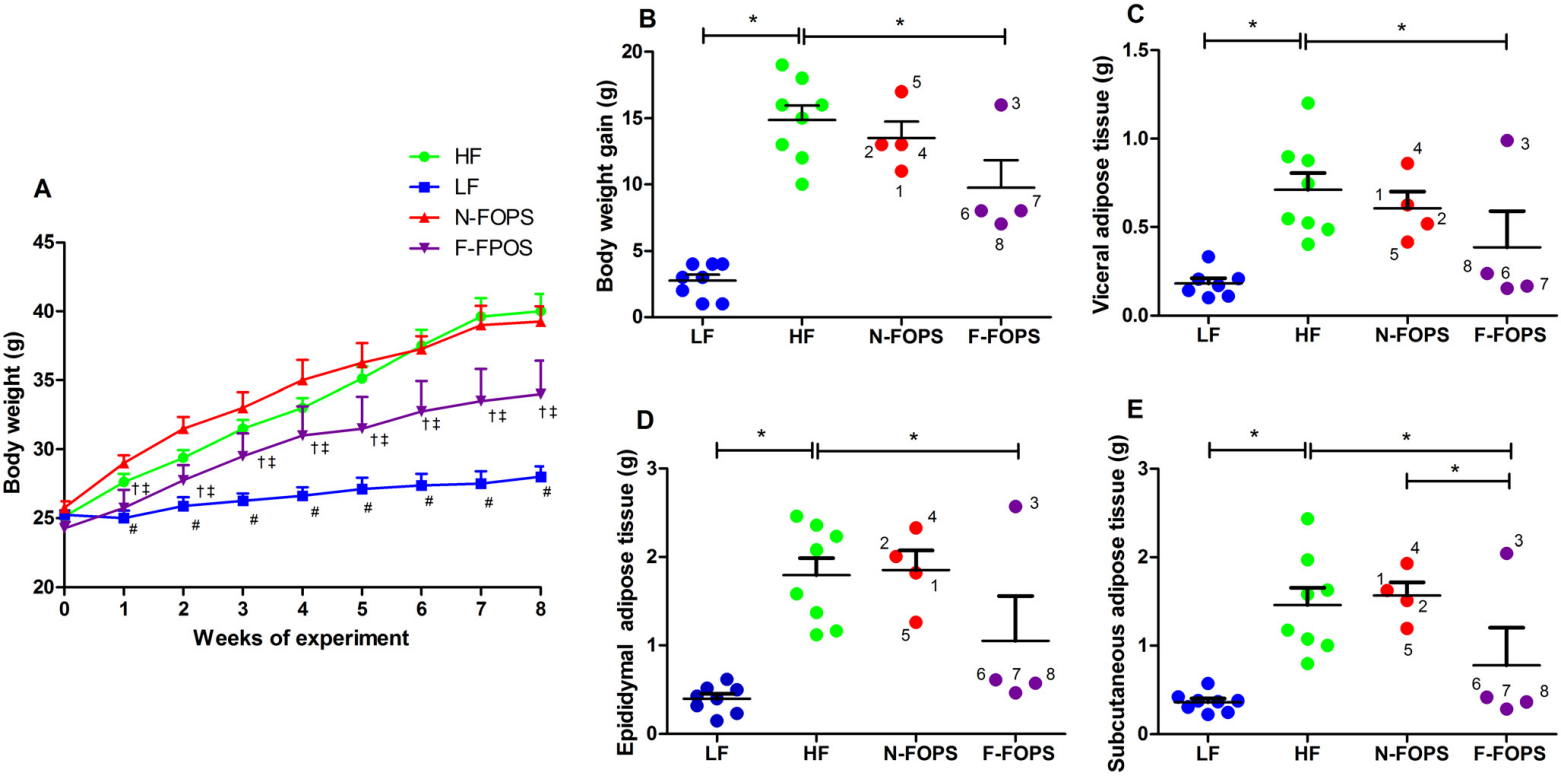
Body and adipose tissue weights in mice after 8 weeks of feeding. Body weight (A), body weight gain (B), and visceral (C), epididymal (D), and subcutaneous (E) adipose tissue weights; data are presented as mean ± SEM; n = 8 mice/group for HF and LF; n = 4 mice/group for N-FOPS and F-FOPS; mouse numbers are shown for FOPS mice to track individual mice across figures; * indicates a significant difference between the indicated treatments using Bonferroni’s multiple comparison test with the following comparisons: LF vs. HF; HF vs. N-FOPS; HF vs. F-FOPS; N-FOPS vs. F-FOPS; #, p<0.05 vs. HF; †, p<0.05 vs. HF; ‡, p<0.05 vs. N-FOPS.

No significant differences in feed intake were observed between HF- and FOPS-fed mice (S1 Fig). However, compared to HF-fed controls, F-FOPS mice experienced a significantly slower rate of weight gain during the eight-week feeding study (Fig 3A) and ultimately gained less weight (Fig 3B). F-FOPS mice also experienced a significant reduction in epididymal, subcutaneous, and visceral adipose tissue weights compared with the HF-fed control mice (Fig 3C–3E). Remarkably, three mice (6, 7, and 8) within the F-FOPS group were found to consistently have adipose tissue weights similar to those of LF-fed mice, while one F-FOPS mouse (3) had adipose tissue weights consistently similar to those of N-FOPS and HF-fed mice. In contrast to F-FOPS mice, body weight gain and adipose tissue weights of the N-FOPS mice mirrored those of the HF-fed control mice. HF feeding also significantly elevated plasma cholesterol and triacylglycerol levels compared to LF-fed mice, but FOPS feeding did not improve the concentrations of these lipids (S2 Fig). No differences in stool consistency were observed among all mice on study (data not shown).

### Mice that Were Capable of Fermenting FOPS Experienced Decreases in Fasting Plasma Glucose and Insulin Levels

**Fig 4:**
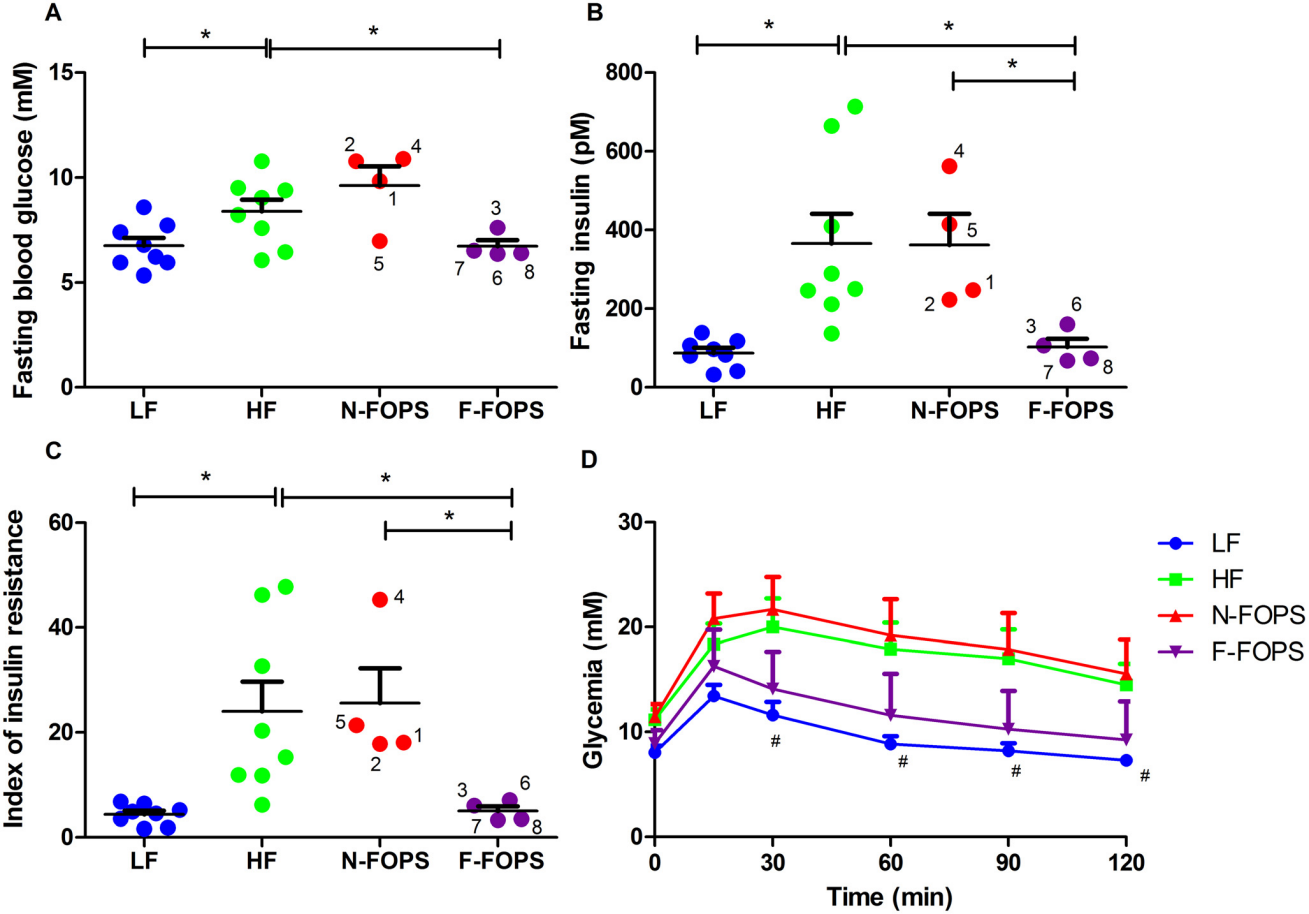
Intraperitoneal glucose tolerance test in mice after 7 weeks of feeding. Fasting glycemia (A), fasting insulinemia (B), index of insulin resistance (C), and glucose tolerance curve (D); data are presented as mean ± SEM; n = 8 mice/group for HF and LF; n = 4 mice/group for N-FOPS and F-FOPS; mouse numbers are shown for FOPS mice to track individual mice across figures; * indicates a significant difference (p < 0.05) between the indicated treatments using Bonferroni’s multiple comparison test with the following comparisons: LF vs. HF; HF vs. N-FOPS; HF vs. F-FOPS; F-FOPS vs. Y-FOPS; #, p<0.05 vs. HF.

As expected, HF feeding increased fasting bood glucose and insulin levels, as well as elevated the index of insulin resistance and impaired glucose tolerance compared with LF feeding (Fig 4), indicating that metabolic aberrations related to impaired glucose tolerance existed in HF-fed control mice. F-FOPS mice experienced significant improvements in both fasting glucose and insulin levels, which were accompanied by a 79% reduction in the index of insulin resistance compared with HF-fed mice. In contrast to weight gain and adipose tissue weight observations, mouse 3 clustered with the other F-FOPS mice, showing similar glycemic and insulinemic profiles to mice 6, 7, and 8. No significant improvements in metabolic parameters were observed for N-FOPS mice compared to HF-fed controls.

### Plasma Peptide Hormone Levels Were Improved in Mice that Fermented FOPS

**Fig 5:**
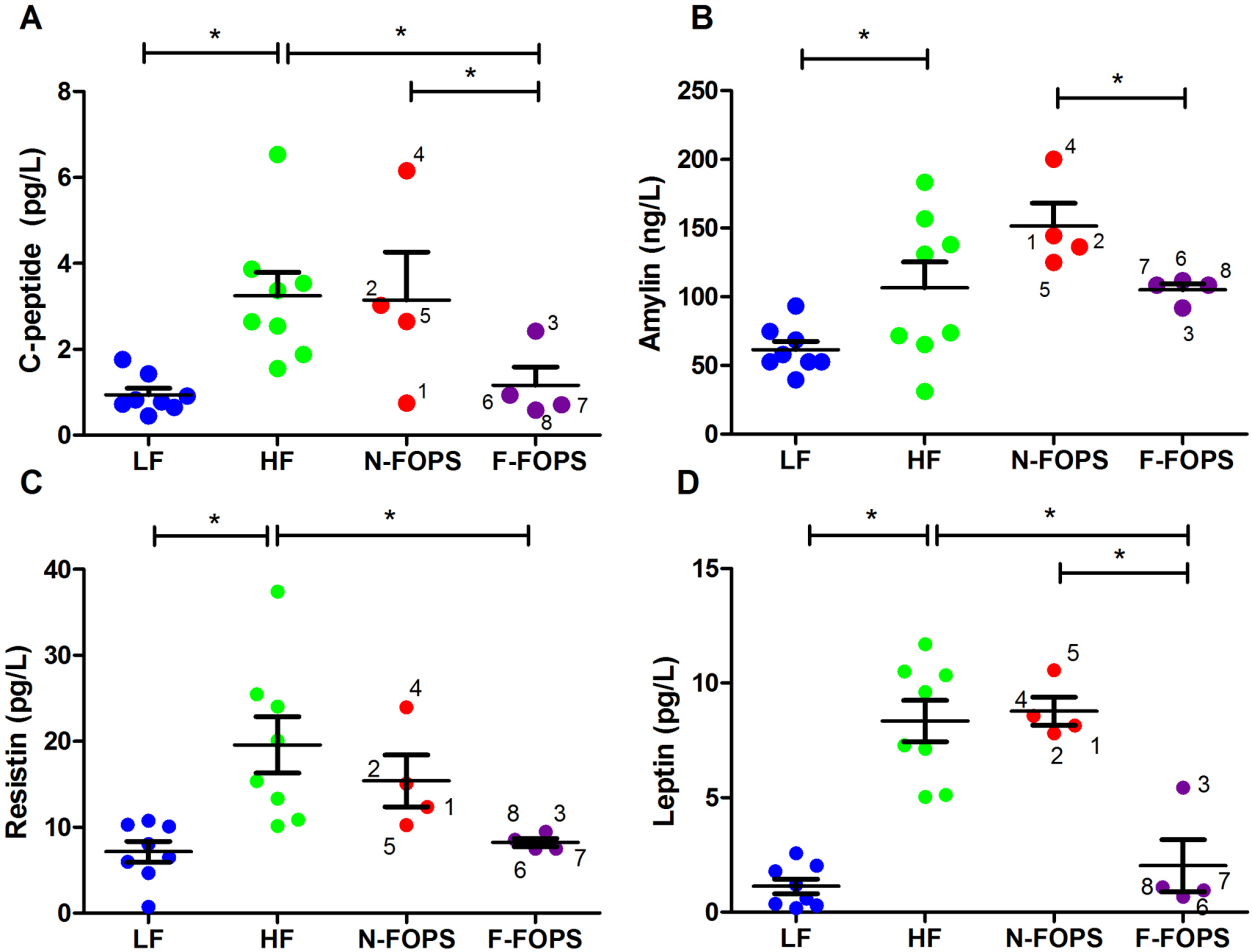
Plasma C-peptide and selected hormone concentrations in mice after 8 weeks of feeding. C-peptide (A), amylin (B), resistin (C), and leptin (D); data are presented as mean ± SEM; n = 8 mice/group for HF and LF; n = 4 mice/group for N-FOPS and F-FOPS; mouse numbers are shown for FOPS mice to track individual mice across figures; * indicates a significant difference (p < 0.05) between the indicated treatments using Bonferroni’s multiple comparison test with the following comparisons: LF vs. HF; HF vs. N-FOPS; HF vs. F-FOPS; N-FOPS vs. F-FOPS.

As expected, HF feeding increased plasma C-peptide and amylin concentrations compared to LF-fed mice (Fig 5A and 5B). Consistent with our observations for insulin levels and the ipGTT, F-FOPS mice experienced a significant reduction in plasma C-peptide concentrations compared to both HF-fed controls and N-FOPS mice (Fig 5A). Plasma levels of the peptide hormone amylin (secreted from pancreatic β-cells) were significantly improved in F-FOPS mice compared to N-FOPS mice (Fig 4B).

The peptide hormones resistin and leptin, which originate from adipose tissue, were also elevated in HF-fed control mice, but were significantly reduced in F-FOPS mice (Fig 5C and 5D). Moreover, leptin levels were also significantly decreased in F-FOPS mice compared with N-FOPS mice. Mouse 3 in the F-FOPS group showed separation from mice 6, 7, and 8 for leptin but not for resistin.

### FOPS Feeding Resulted in Divergent Fecal Microbiota Compositions

**Fig 6:**
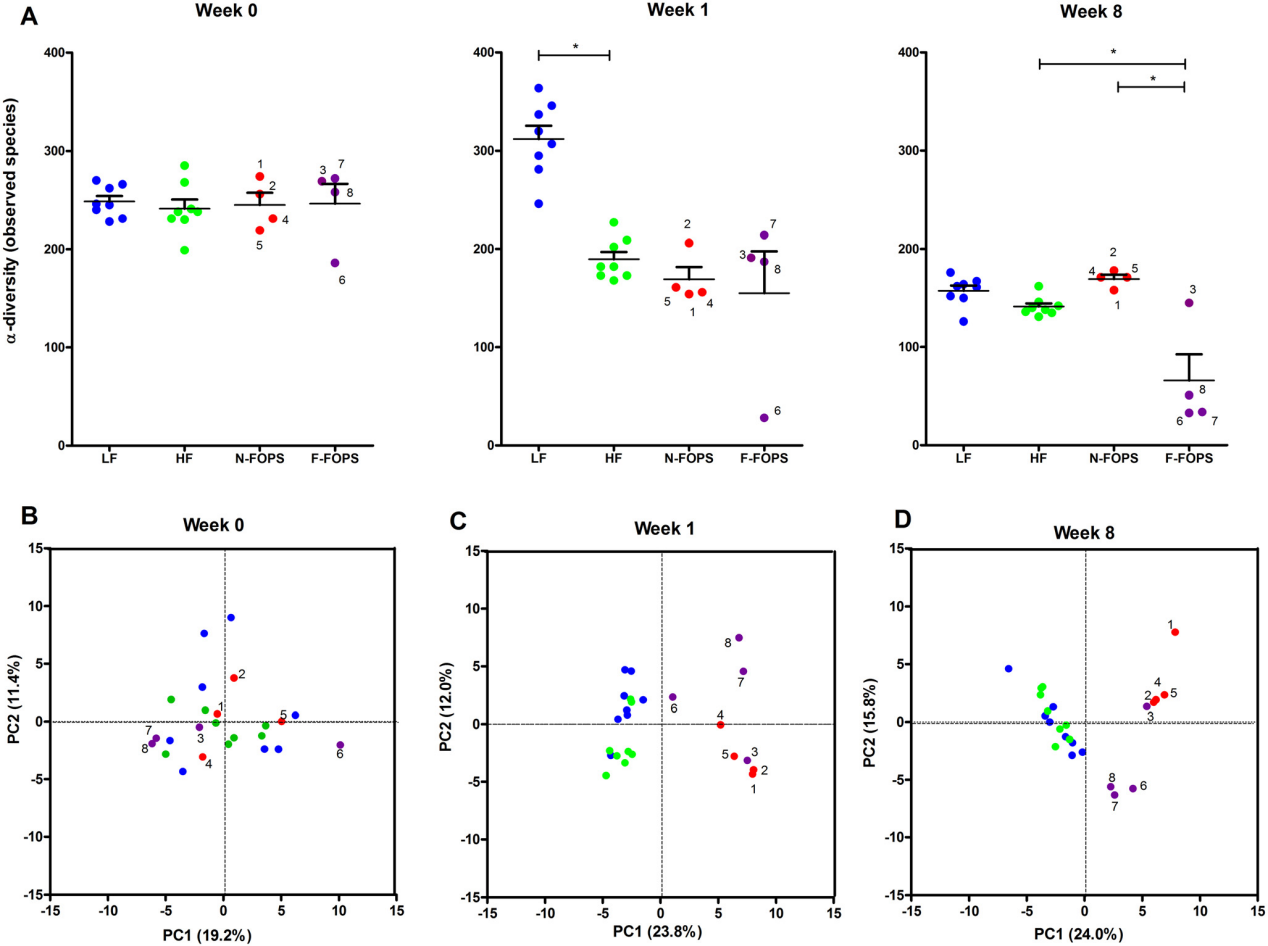
Diversity and principal components analysis of fecal bacterial communities in mice at 0, 1, and 8 weeks of feeding. α-Diversity (A) and principal components analysis of OTU abundances (B-D); data are presented in (A) as the mean ± SEM; n = 8 mice/group for HF and LF; n = 4 mice/group for N-FOPS and F-FOPS. (B-D) blue dots represent LF, green dots represent HF, red dots represent N-FOPS, purple dots represent F-FOPS; mouse numbers are shown for FOPS mice to track individual mice across figures; *indicates a significant difference (p < 0.05) between the indicated treatments using Bonferroni’s multiple comparison test with the following comparisons: LF vs. HF; HF vs. N-FOPS; HF vs. F-FOPS; N-FOPS vs. F-FOPS.

To explore if the metabolic benefits induced by FOPS supplementation, and especially the inter-individual variability in these effects, were associated with shifts in the gut microbiota, we characterized the fecal microbiota by 16S rRNA tag Illumina sequencing. α-Diversity of the fecal microbiota significantly decreased in HF- and FOPS-fed mice at week 1 compared with the LF group (Fig 6A). At week 8, the α-diversity of the mice in the F-FOPS group further decreased compared with the HF and N-FOPS groups. Principal component analysis (PCA) based on OTU abundance showed no clustering by treatment group at baseline (Fig 6B), whereas principal component 1 separated FOPS-fed mice from HF- and LF-fed mice at week 1 (Fig 6C)—a separation that persisted after 8 weeks of feeding (Fig 6D). F-FOPS mice 6, 7, and 8 clustered separately from the other mice, while F-FOPS mouse 3 was more similar to the N-FOPS mice.

**Fig 7:**
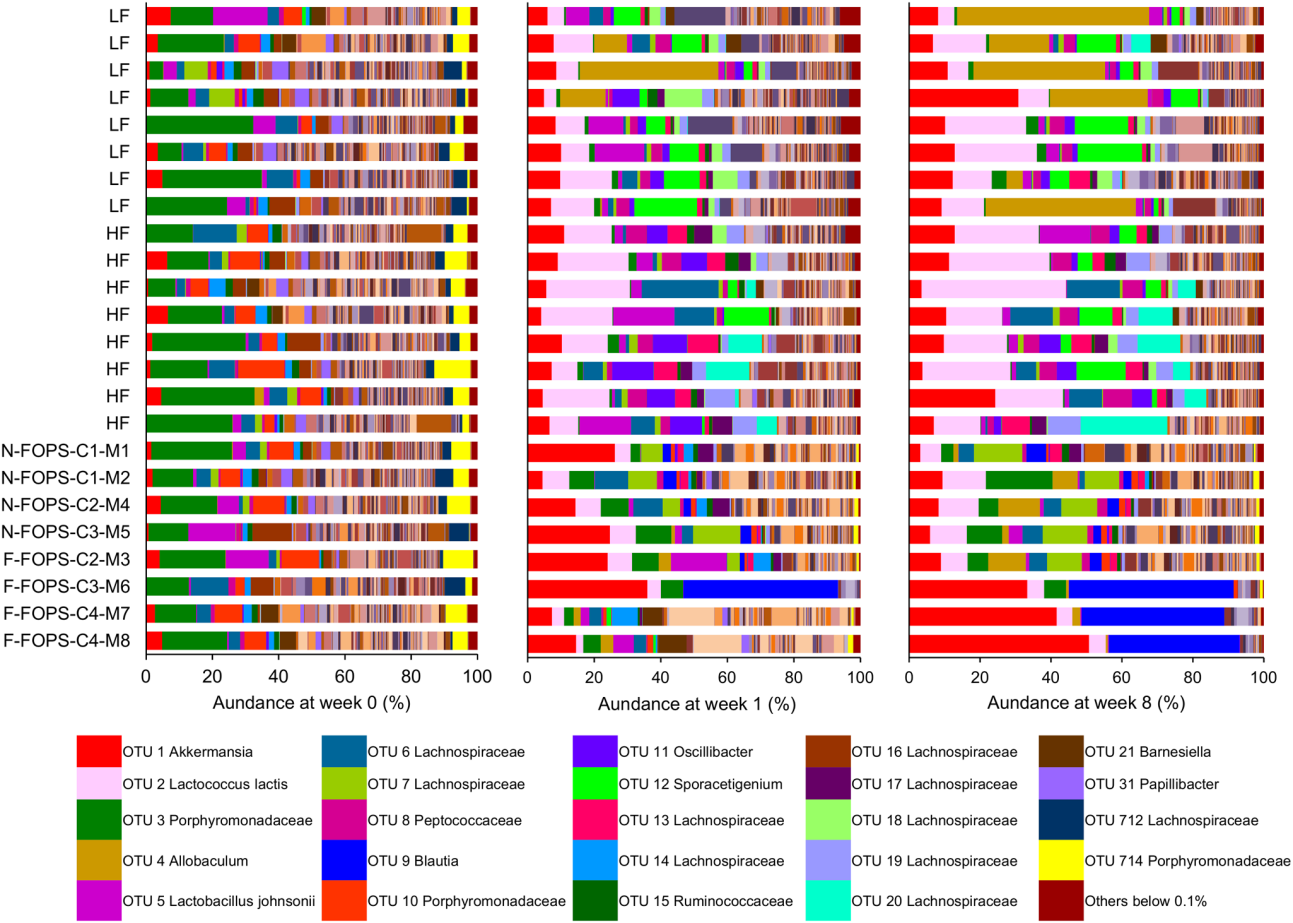
Fecal microbiota compositions based on OTU abundance for each mouse after 0, 1, and 8 weeks of feeding. For FOPS mice, C1, C2, etc. indicate cage 1, cage 2, etc., while M1, M2, etc. indicate mouse 1, mouse 2, etc.; the color key corresponds to the 25 most abundant OTUs across all time points.

At week 0, all mice had similar baseline microbial compositions (Fig 7) that were dominated by members of the Firmicutes and Bacteroidetes phyla (S3 Table). Because of the disparate responses to FOPS among the FOPS fed mice, we were particularly interested in assessing differences between N-FOPS and F-FOPS mice at this time point. At the phylum level, N-FOPS and F-FOPS mice had significantly higher proportions of Proteobacteria (S3 Table). At the OTU level, N-FOPS mice harbored significantly more of one OTU belonging to the Lachnospiraceae family (OTU 64) compared with the F-FOPS mice, and the F-FOPS mice had significantly higher *Acetivibrio* (OTU 259) levels compared to N-FOPS mice (S4 Table).

Many changes could already be noted by week 1, indicating the rapid effect of diet on altering the microbiota composition (Fig 7). The F-FOPS and N-FOPS mice also became distinguishable from one another, with a number of significant differences at the phylum (S3 Table) and OTU (S5 Table) levels. In particular, 19 OTUs belonging to the Lachnospiraceae (OTUs 7, 32, 39, 42, 43, 66, 69, 99, 111, 185, 186, 209, 699, and 712), Ruminococcaceae (OTU 104, and 167), and Erysipelotrichaceae (OTU 159) families and *Dorea* (OTU 75) and *Marvinbryantia* (OTU 155) genera were significantly higher in N-FOPS mice, while 3 OTUs belonging to the Ruminococcaceae family (OTU 225) and *Bifidobacterium* (OTU 22) and *Acetivibrio* (OTU 27) genera were significantly higher in F-FOPS mice.

The most remarkable change in microbiota composition over the course of the study was the substantial increase in OTUs belonging to the *Blautia* and *Akkermansia* genera that occurred in three of the F-FOPS mice at week 8 (Fig 7). Combined, these two OTUs represented approximately 80% of the fecal microbiota in F-FOPS mice 6, 7, and 8 and explain the substantial decrease in α-diversity in these mice. Notably, these are the same three mice that clustered together on the PCA plots (Fig 6B–6D). In contrast, mouse 3 (also in the F-FOPS group as it had an enlarged cecum), did not harbor elevated *Blautia* and *Akkermansia* at the end of the study.

At week 8, F-FOPS mice had significantly elevated Verrucomicrobia compared with the HF and N-FOPS groups (S3 Table). This was accompanied by a significant decrease in Firmicutes compared with the HF-fed mice. In contrast, the N-FOPS mice had significantly elevated Bacteroidetes compared with the HF mice. Both N-FOPS and F-FOPS had significantly lower proportions of many OTUs belonging to the Peptococcaceae (OTU 8), Lachnospiraceae (OTU 19, 25, 46, 53, 76, 93, 96, 179), and Ruminococcaceae (OTU 129, 638) families, *Oscillibacter* (OTU 11), *Anaerotruncus* (OTU 51, 141), *Coprobacillus* (OTU 84), *Coprococcus* (OTU 127), *Dorea* (OTU 153, 169) genera, and *Lactococcus lactis* (OTU 2, 695) species compared with the HF-fed mice, but had higher abundances of one OTU belonging to the Porphyromonadaceae (OTU 10) family and one OTU belonging to the *Anaerotruncus* (OTU 62) genus (S6 Table). Only 4 OTUs were significantly elevated in the F-FOPS mice compared with both the HF-fed mice and the N-FOPS mice. These corresponded to OTUs belonging to the Ruminococcaceae (OTU 89) family and the *Akkermansia* (OTU 1), *Blautia* (OTU 9), and *Anaerotruncus* (OTU 190) genera.

### Microbial Composition and Fermentation Correlated with Improvements in Metabolic Parameters

**Fig 8:**
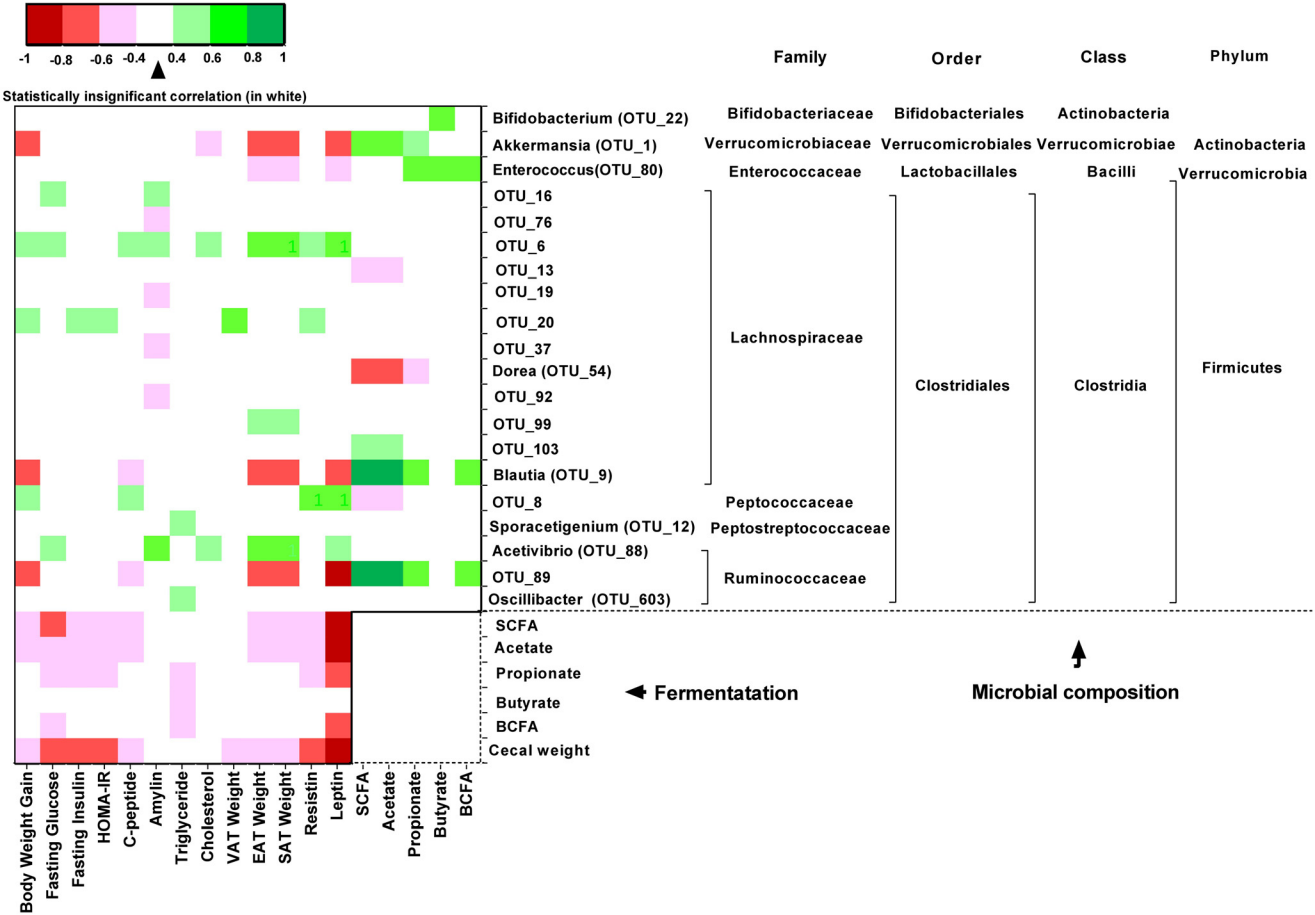
Correlations between relative abundance of fecal bacterial OTUs and short and branched chain fatty acids with output variables in HF and FOPS mice. Only statistically significant correlations are shaded (p<0.05).

Body weight gain, leptin concentration, and EAT and SAT weights were all negatively correlated with *Akkermansia*, *Blautia* and OTU 89 (a member of the *Ruminococcaceae* family; Fig 8); plasma cholesterol levels were also negatively correlated with *Akkermansia* abundance. There were positive correlations between SCFA production and the abundance of *Akkermansia*, *Blautia*, and OTU 89. OTU 6 and, in most cases, OTU 20 (both members of the *Lachnospiraceae* family) were positively correlated with negative outcomes: body weight gain, fasting glucose, C-peptide, amylin, plasma cholesterol, leptin, resistin, and the weight of EAT and SAT. Markers of microbial fermentation of FOPS, including total SCFA, acetate, and cecal weight were negatively correlated with many host metabolic markers, including body weight gain, fasting glucose, fasting insulin, index of insulin resistance, C-peptide, adipose tissue weights, resistin, and leptin. Notably, propionate was significantly correlated with markers of glucose homeostasis and insulin resistance but not body weight gain and adipose tissue weights.

## Discussion

In this study, we investigated how FOPS affected body weight gain, adipose tissue weights, markers of metabolic syndrome, and gut microbiota composition in mice fed a HF diet. We observed disparate metabolic responses to the FOPS diet intervention that were strongly linked to FOPS-induced changes in gut microbiota composition and function (fermentation to SCFA). Enhanced cecal fermentation with associated metabolic improvements was evident in some mice (F-FOPS), while other mice showed normal cecal fermentation and no metabolic improvements compared with mice in the HF group (N-FOPS).

Significantly lower fasting blood glucose and insulin levels were observed for F-FOPS mice compared with HF-fed control mice. These improvements in glucose homeostasis in the F-FOPS mice might be due to enhanced SCFA production from the FOPS fermentation as butyrate and propionate have been shown to protect against diet-induced obesity as well as reduce fasting insulin and leptin levels [31]. In particular, F-FOPS mice exhibited substantial increases in cecal propionate production, which were significantly correlated with reductions in fasting glucose and insulin levels, the index of insulin resistance, and plasma leptin levels. Propionate may limit weight gain by partially suppressing food intake [31]; however, in the present study, we observed no effects on food intake during FOPS feeding. SCFA can also limit weight gain through increased energy expenditure [32] and improve host metabolism by stimulating secretion of the incretin hormone glucagon-like peptide 1 and activating the SCFA receptor GPR43 [33]. Together, these observations indicate that F-FOPS mice may have experienced improvements in glucose metabolism via enhanced SCFA production.

F-FOPS mice also showed significant reduction in body weight gain compared with HF-fed mice. However, the mechanism responsible for weight gain reduction appears to be different from that responsible for improvements in glucose tolerance, as one F-FOPS mouse did not experience a reduction in fat mass and body weight gain. Interestingly, the three F-FOPS mice with the greatest improvements in body weight gain, adipose weights, and leptin experienced dramatic shifts in the composition of their gut microbiota, especially in blooms of the genera *Akkermansia* and *Blautia*. In contrast, these changes were not observed in mice that did not exhibit these improvements, suggesting that these two bacterial groups may be involved in mediating these specific benefits. Recent studies have demonstrated an inverse association between *Akkermansia* and *Blautia* and markers of obesity-related metabolic disorders [34–36]. The intake of fructans by obese mice increased the abundance of *Akkermansia* by ~100 fold with a concomitant reduction in fat mass gain and adipose tissue inflammation [37]. *Akkermansia* may affect host metabolism through the enhancement of gut barrier function or production of endocannabinoids that control inflammation [38]. In the case of *Blautia*, diet-induced obesity strongly reduces the abundance of this genus in mice [39]. Another study suggested that the prevention of obesity and insulin resistance in HF-fed rats with berberine, a yellow pigment found in some plant tissues, may be partially mediated by *Blautia* [40]. Given that obesity is linked to leptin resistance and that leptin is primarily involved in energy expenditure [41], the significant decrease in plasma leptin levels in F-FOPS mice (especially in mice 6, 7, and 8) also suggests enhanced leptin sensitivity in these mice, which might be responsible for their limited weight gain [41]. Plasma leptin concentration was also negatively correlated with *Akkermansia* and *Blautia*. Together, these findings suggest the importance of *Akkermansia* and *Blautia* in the metabolic improvements related to weight reduction with FOPS.

Our observation of FOPS fermenter and non-fermenter mice was an unexpected finding. We did not anticipate that inter-individual responsiveness during FOPS feeding would necessitate dividing the mice into two groups, which created a low number of mice and considerable variation in some of the responses. Despite these limitations, we were still able to observe remarkable differences in many phenotypes, including body weight gain, adipose tissue weights, and blood glucose tolerance. These observations clearly support the conclusion that the metabolic improvements related to glucose homeostasis induced by FOPS are the consequence of colonic fermentation of FOPS and production of SCFA, while the metabolic improvements related to weight reduction may be linked to the unique gut microbiota composition, including high proportions of *Akkermansia* together with *Blautia*.

It is unclear why mice in the FOPS group responded so differently to the treatment. While the phenomenon of responders versus non-responders to fiber and prebiotic interventions has been described in human studies [42, 43], it is less common in mouse studies (although not unheard of [44]) as genetics and environmental factors are better controlled. Once confirming that the differences in FOPS responsiveness were not due to cage effects, we hypothesized that the four mice in the F-FOPS group may have harbored different gut microbiota profiles at baseline that enabled them to better utilize the FOPS substrate as compared to the N-FOPS mice. As mentioned, FOPS contains unusual linkages, including (1, 2) and (1, 3)-linkages connecting branched chain xylose and arabinose, which may have required the presence of uncommon carbohydrate-active enzymes to initiate fermentation [45]. Bacteria capable of expressing such enzymes may allow for the fermentation of FOPS and subsequent changes in the microbiota by cross feeding. Diversity and PCA plots suggested that the overall community structures between N-FOPS and F-FOPS at baseline were similar; however, there were subtle differences that may be meaningful. For instance, *Acetivibrio cellulolyticus*, the genus of which was elevated in the F-FOPS mice, can secrete an α-L-arabinofuranosidase [46], a major structural component of FOPS. Alternatively, functional differences, not detectable through 16S rRNA community profiling, may have existed between the FOPS-fed mice that fermented FOPS and those that did not.

Another possibility is that members within the microbial communities of individual mice adapted to metabolize the unusual linkages in FOPS. Because such an evolutionary process would be driven by natural selection after random mutations, it would be, to a large degree, stochastic and dependent on time, thereby causing variation within mice [47]. It is therefore possible that the study duration may have not been long enough to allow adaptation to occur in all mice.

Taken together, we observed heterogeneous metabolic adaptation of mice to a FOPS intervention. Our findings provide new evidence for modulating the gut microbiota through dietary fiber treatment and indicate its contribution to the improvement of host metabolism. Although we cannot provide a mechanistic explanation for the different responses to FOPS treatment, our findings provide strong evidence that the metabolic improvements related to glucose homeostasis induced by FOPS in obese mice are the consequence of colonic fermentation of FOPS and production of SCFA, while the metabolic improvements related to weight reduction might be linked to increases of beneficial microbes (e.g., the genus *Akkermansia*). Our findings strongly support a causal role of gut bacteria in mediating the health benefits of dietary fiber. In addition, our findings provide a potential explanation for why the observed metabolic effects of dietary fiber are often inconsistent in human intervention trials. As humans harbor microbial communities that are much more individualized than those of experimental mice, they may therefore differ substantially in their ability to utilize specific fibers and provide benefits.

## Supporting Information

S1 Fig Average food consumption by cage during the 8 week feeding study. Data are presented as individual observations with n = 2 mice/cage. *indicates a significant difference between the indicated treatments (p<0.05).
(DOCX)

S2 Fig Plasma cholesterol (A) and triglyceride (B) levels after 8 weeks of feeding. Data are presented as mean±SEM; n = 8 mice/group for HF and LF; n = 4 mice/group for N-FOPS and F-FOPS; mouse numbers are shown for FOPS mice to track individual mice across figures; *indicates a significant difference (p < 0.05) between the indicated treatments using Bonferroni’s multiple comparison test with the following comparisons: LF vs. HF; HF vs. N-FOPS; HF vs. F-FOPS; N-FOPS vs. F-FOPS.
(DOCX)

S1 Table Composition of FOPS. Values are reported as mean±standard deviation (n = 2); ND, none detected; HMF, hydroxymethylfurfural.
(DOCX)

S2 Table Nutrient composition of diets fed to male C57BL/6J mice. LF, low fat; HF, high fat diet; FOPS, high fat diet with FOPS; LF (D12450K) and HF formulation were based on D12492 from Research Diets; crude FOPS preparation is the final product obtained after freeze-drying process and contains a portion of FOPS.
(DOCX)

S3 Table Abundance of phyla by treatment group. #/N, proportion of mice with detectable levels of that particular phylum; significant ANOVA models (p<0.05) and Bonferroni contrasts (p<0.0125) are bolded.
(XLSX)

S4 Table Abundance of OTUs (% of total sequences) by treatment group at week 0. #/N, proportion of mice with detectable levels of that particular OTU; significant ANOVA models (p<0.05) and Bonferroni contrasts (p<0.0125) are bolded; OTUs for which all mice across all time points had abundances <0.1% were combined into "Others below 0.1%".
(XLSX)

S5 Table Abundance of OTUs (% of total sequences) by treatment group at week 1. #/N, proportion of mice with detectable levels of that particular OTU; significant ANOVA models (p<0.05) and Bonferroni contrasts (p<0.0125) are bolded; OTUs for which all mice across all time points had abundances <0.1% were combined into "Others below 0.1%".
(XLSX)

S6 Table Abundance of OTUs (% of total sequences) by treatment group at week 8. #/N, proportion of mice with detectable levels of that particular OTU; significant ANOVA models (p<0.05) and Bonferroni contrasts (p<0.0125) are bolded; OTUs for which all mice across all time points had abundances <0.1% were combined into "Others below 0.1%".
(XLSX)
