# Supplementary material for: Disparate Metabolic Responses in Mice Fed a High-Fat Diet Supplemented with Maize-Derived Non-Digestible Feruloylated Oligo- and Polysaccharides Are Linked to Changes in the Gut Microbiota
Source: PLoS One. 2016 Jan 5;11(1):e0146144. doi: 10.1371/journal.pone.0146144 (PMC4701460; doi:10.1371/journal.pone.0146144)
Supplement: S1 Fig — Data are presented as individual observations with n = 2 mice/cage. *indicates a significant difference between the indicated treatments (p<0.05). (DOCX) [file pone.0146144.s001.docx]

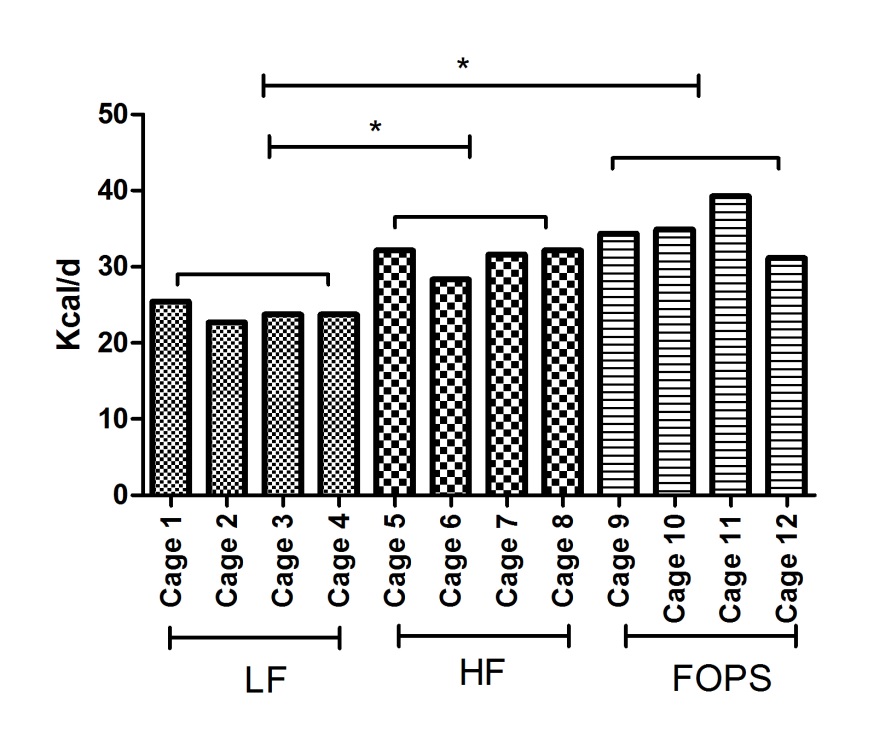


**S1 Fig. Average food consumption by cage during the 8 week feeding study.** Data are presented as individual observations with n=2 mice/cage. *indicates a significant difference between the indicated treatments (p<0.05).
