# Supplementary material for: Disparate Metabolic Responses in Mice Fed a High-Fat Diet Supplemented with Maize-Derived Non-Digestible Feruloylated Oligo- and Polysaccharides Are Linked to Changes in the Gut Microbiota
Source: PLoS One. 2016 Jan 5;11(1):e0146144. doi: 10.1371/journal.pone.0146144 (PMC4701460; doi:10.1371/journal.pone.0146144)
Supplement: S2 Fig — Data are presented as mean±SEM; n = 8 mice/group for HF and LF; n = 4 mice/group for N-FOPS and F-FOPS; mouse numbers are shown for FOPS mice to track individual mice across figures; *indicates a significant difference (p < 0.05) between the indicated treatments using Bonferroni’s multiple comparison test with the following comparisons: LF vs. HF; HF vs. N-FOPS; HF vs. F-FOPS; N-FOPS vs. F-FOPS. (DOCX) [file pone.0146144.s002.docx]

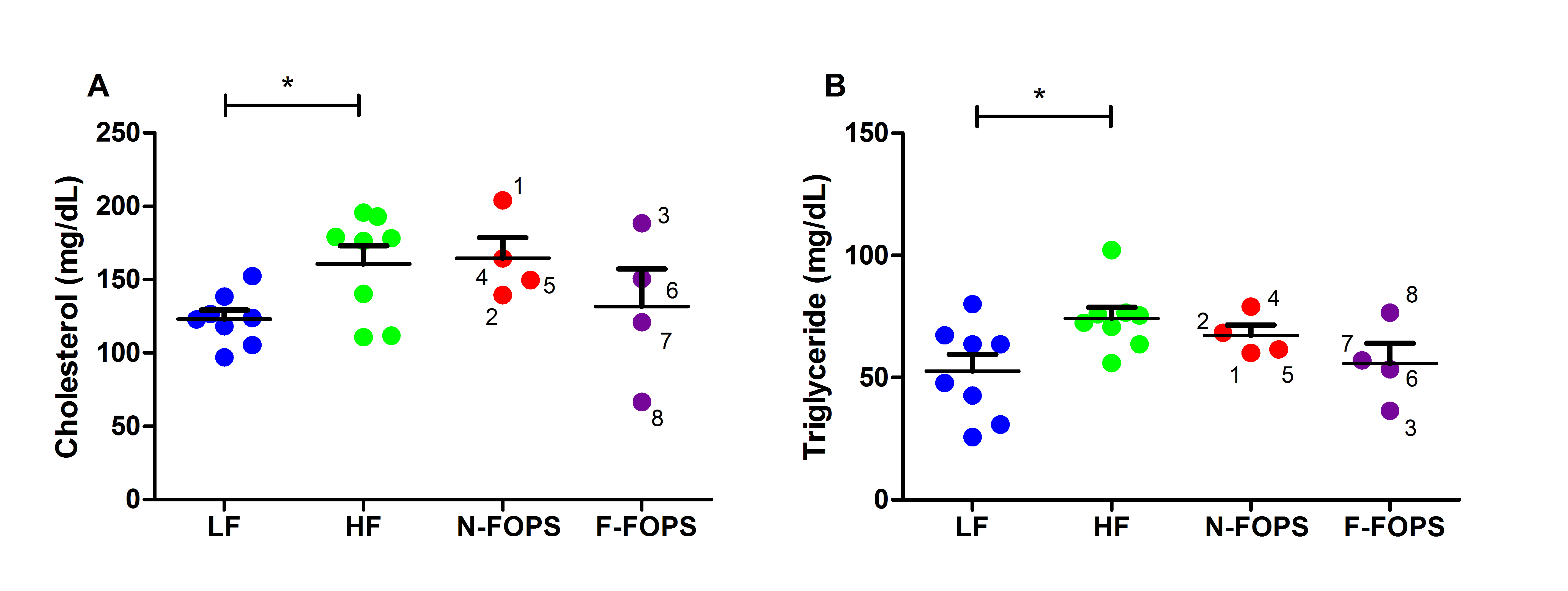


**S2 Fig. Plasma cholesterol (A) and triglyceride (B) levels after 8 weeks of feeding.** Data are presented as mean±SEM; n=8 mice/group for HF and LF; n=4 mice/group for N-FOPS and F-FOPS; mouse numbers are shown for FOPS mice to track individual mice across figures; *indicates a significant difference (p < 0.05) between the indicated treatments using Bonferroni’s multiple comparison test with the following comparisons: LF vs. HF; HF vs. N-FOPS; HF vs. F-FOPS; N-FOPS vs. F-FOPS.
