# Supplementary material for: Disparate Metabolic Responses in Mice Fed a High-Fat Diet Supplemented with Maize-Derived Non-Digestible Feruloylated Oligo- and Polysaccharides Are Linked to Changes in the Gut Microbiota
Source: PLoS One. 2016 Jan 5;11(1):e0146144. doi: 10.1371/journal.pone.0146144 (PMC4701460; doi:10.1371/journal.pone.0146144)
Supplement: S1 Table — Values are reported as mean±standard deviation (n = 2); ND, none detected; HMF, hydroxymethylfurfural. (DOCX) [file pone.0146144.s003.docx]

**S1 Table. Composition of FOPS.** Values are reported as mean±standard deviation (n=2); ND, none detected; HMF, hydroxymethylfurfural.

| Component | Composition |
| --- | --- |
| Total carbohydrate (%) | 74.4 |
| FOPS (%) | 59.1 |
| Arabinan (%) | 10.13±0.19 |
| Xylan (%) | 33.55±1.29 |
| Mannan (%) | 0.30±0.00 |
| Galactan (%) | 4.71±0.21 |
| Glucan (%) | 4.44±0.56 |
| Esterified ferulate (%) | 4.13±0.03 |
| Uronic acid | 1.88±0.08 |
| Free monosaccharides (%) | 3.44±0.09 |
| Arabinose (%) | 1.28±0.02 |
| Xylose (%) | 1.06±0.06 |
| Mannose (%) | ND |
| Galactose (%) | 0.53±0.01 |
| Glucose (%) | 0.58±0.03 |
| Starch (%) | 15.98±0.51 |
| Free ferulic acid (%) | ND |
| Protein (%) | 2.62±0.05 |
| Furfural (%) | ND |
| HMF (%) | ND |
| Moisture content (%) | 8.77 |
