# Supplementary material for: Disparate Metabolic Responses in Mice Fed a High-Fat Diet Supplemented with Maize-Derived Non-Digestible Feruloylated Oligo- and Polysaccharides Are Linked to Changes in the Gut Microbiota
Source: PLoS One. 2016 Jan 5;11(1):e0146144. doi: 10.1371/journal.pone.0146144 (PMC4701460; doi:10.1371/journal.pone.0146144)
Supplement: S2 Table — LF, low fat; HF, high fat diet; FOPS, high fat diet with FOPS; LF (D12450K) and HF formulation were based on D12492 from Research Diets; crude FOPS preparation is the final product obtained after freeze-drying process and contains a portion of FOPS. (DOCX) [file pone.0146144.s004.docx]

**S2 Table. Nutrient composition of diets fed to male C57BL/6J mice.** LF, low fat; HF, high fat diet; FOPS, high fat diet with FOPS; LF (D12450K) and HF formulation were based on D12492 from Research Diets; crude FOPS preparation is the final product obtained after freeze-drying process and contains a portion of FOPS.

| Diet | LF | HF | FOPS |
| --- | --- | --- | --- |
| ***Ingredient%*** | |  |  |
| Casein | 19.0 | 25.8 | 25.1 |
| L-Cystine | 0.284 | 0.388 | 0.381 |
| Corn Starch | 52.1 | 0 | 0 |
| Maltodextrin 10 | 14.2 | 16.2 | 14.4 |
| Sucrose | 0 | 8.89 | 8.73 |
| Cellulose | 4.742 | 6.46 | 1.32 |
| Crude FOPS | 0 | 0 | 8.51 |
| Lard | 1.90 | 31.7 | 31.1 |
| Soybean Oil | 2.37 | 3.2 | 3.17 |
| Mineral Mix S10026 | 0.948 | 1.29 | 1.27 |
| Dicalcium Phosphate | 1.23 | 1.69 | 1.65 |
| Calcium Carbonate | 0.521 | 0.711 | 0.698 |
| Potassium Citrate, 1 H_2_O | 1.56 | 2.13 | 2.09 |
| Vitamin Mix V10001 | 0.948 | 1.292 | 1.27 |
| Choline Bitartrate | 0.190 | 0.258 | 0.254 |
| Red Dye #40, FD&C | 0.00237 | 0 | 0.00635 |
| Blue Dye #1, FD&C | 0.00237 | 0.00646 | 0 |
| ***Weight%*** |  |  |  |
| Protein | 19.2 | 23.1 | 22.7 |
| Carbohydrate | 67.3 | 26.3 | 25.9 |
| FOPS | 0 | 0 | 5 |
| Fat | 4.30 | 35.2 | 34.6 |
| Fiber | 4.70 | 6.50 | 6.30 |
| ***kcal%*** |  |  |  |
| Protein | 20 | 18 | 18 |
| Carbohydrate | 70 | 20 | 20 |
| Fat | 10 | 62 | 62 |
| Total | 100 | 100 | 100 |
| kcal/gm | 3.90 | 5.10 | 5.10 |
